# The Effect of B_2_O_3_ Doping on the Properties of Electrical and Thermal Conductivity for SnO_2_ Varistors

**DOI:** 10.3390/ma18071399

**Published:** 2025-03-21

**Authors:** Siqiao Gong, Hongfeng Zhao

**Affiliations:** The Wind Solar Storage Division, State Key Laboratory of Control and Simulation of Power System and Generation Equipment, School of Electrical Engineering, Xinjiang University, Urumqi 830046, China; 13699353694@163.com

**Keywords:** varistors, thermal conductivity, electrical properties, impurities

## Abstract

This study investigates SnO_2_-based varistors in the SnO_2_-Co_3_O_4_-Cr_2_O_3_-Nb_2_O_5_ system with varying B_2_O_3_ doping concentrations to optimize both electrical properties and thermal conductivity. The experimental formulation involved doping B_2_O_3_ with fixed concentration ratios of Co_3_O_4_, Cr_2_O_3_, and Nb_2_O_5_ (ranging from 0 mol% to 0.35 mol%), and the microstructure, electrical properties, and thermoelectric coefficient of the samples were measured in order to identify the optimal doping proportion. The varistor doped with 0.25 mol% B_2_O_3_ exhibited optimal performance, demonstrating a maximum voltage gradient of 525 V/mm, a minimum leakage current density of 11.2 μA/cm^2^, and a peak nonlinear coefficient of 36. Furthermore, the optimized formulation achieved enhanced thermal performance with a maximum thermal conductivity of 6.13 W·m^−1^·K^−1^.

## 1. Introduction

As the construction of ultra-high-voltage (UHV) power grid projects advanced gradually, the voltage levels and transmission capacities of these grids also increased progressively, which in turn led to a higher incidence of lightning-induced tripping. Statistics indicated that in China’s power system, more than 50% of the faults occurring annually were caused by lightning strikes, resulting in the tripping of transmission lines [1,2]. The installation of high-performance surge arresters had been proven to effectively suppress the overvoltage levels in power transmission systems within the limits that the insulation of the power equipment could withstand. This not only enhanced the insulation level of the power system but also simplified the insulation design of the power system, thereby reducing the manufacturing costs and technical difficulties associated with insulating equipment [3].

Currently, zinc oxide (ZnO) and tin oxide (SnO_2_) are the more commonly used metal oxides in surge arresters. Unlike the complex multiphase structure of ZnO varistors, SnO_2_ varistor ceramics possess a single-phase structure. Moreover, the additives in SnO_2_ varistor ceramics exhibit minimal volatilization during the sintering process, resulting in negligible doping losses due to volatilization. This characteristic endows SnO_2_ varistor ceramics with a more uniform microstructure. Consequently, the current flowing through its interior, as well as the corresponding temperature and thermal stress distributions, are more uniform under various operating conditions [4,5,6]. The thermal conductivity of SnO_2_ varistors at the same temperature is almost twice that of ZnO varistors. Compared with the latter, SnO_2_ varistors have better thermal conductivity and high-temperature resistance. Good thermal conductivity can quickly transfer the local temperature of the varistor, improving the safety and stability of the power system. Furthermore, SnO_2_-based piezoresistive ceramics exhibit distinct advantages including a simplified microstructure configuration and enhanced densification characteristics (relative density >98% TD) [7,8].

One study [9] demonstrated that SnO_2_ varistors with optimized grain boundary barrier characteristics can be fabricated through a segregation-induced and acceptor co-doping strategy, thereby achieving enhanced electrical nonlinearity with a nonlinear coefficient (α) of 36 ± 0.5. In the fabrication process of SnO_2_ varistors, the addition of a small amount of CoO/Co_2_O_3_, Nb_2_O_5_, and Cr_2_O_3_ can regulate the amplitude and width of the voltage barriers at the grain boundaries, thereby obtaining samples with different microstructures [10,11].

Another study [12] reported that SnO_2_ varistors containing 1.00 mol% CoO, 0.05 mol% Cr_2_O_3_, and 0.05 mol% Nb_2_O_5_ had a nonlinear coefficient of 41 and a voltage gradient of 400 V/mm. However, a rapid increase in the local temperature of the varistor can cause thermal collapse [13]. The doping of B_2_O_3_ in ZnO varistor formulations facilitates ionic exchange between ZnO grains and additive components at the grain boundaries, thereby effectively enhancing the barrier height ($\varphi_{b}$) while reducing leakage currents [11].

Several studies have reported the use of different nanomaterials for varistors. For example, ZnO-based nanomaterials have been widely studied due to their excellent nonlinear electrical properties [13]. However, SnO_2_-based nanomaterials have gained attention because of their single-phase structure, minimal volatility of additives during sintering, and superior thermal conductivity compared to ZnO varistors. The performance of varistors can be significantly improved by tuning the nanomaterials. Adjusting the grain size, doping concentration, and microstructure can optimize the electrical and thermal properties. For instance, the formation of oxygen vacancies through doping can enhance the nonlinearity coefficient and conductivity [13,14,15].

In order to find a formulation with both good electrical properties and high thermal conductivity, this paper adds 0.05–0.35 mol% B_2_O_3_ to the doping system of SnO_2_-Co_3_O_4_-Cr_2_O_3_-Nb_2_O_5_ described in the literature [10,11,12,16]. Through experimental tests, it analyses the effect of different B_2_O_3_ doping amounts on the microstructure of SnO_2_ varistors and further explores the influence of the microstructure on both the electrical properties and thermal conductivity of SnO_2_ varistors. It also explores how to better improve the performance of piezoresistors by tuning the structure and properties of nanomaterials, e.g., by controlling microstructural factors such as grain size, porosity, and oxygen vacancy concentration.

## 2. Experimental

Samples of SnO_2_ with varied contents of B_2_O_3_-doped varistors were prepared based on the SnO_2_-Co_3_O_4_-Cr_2_O_3_-Nb_2_O_5_ system with the following raw materials: SnO_2_ (98.2% sample purity), Co_3_O_4_ (97.8% sample purity), Cr_2_O_3_ (97.9% sample purity), Nb_2_O_5_ (98.3% sample purity), and B_2_O_3_ (98.4% sample purity). The samples were prepared according to the following ratios (all molar fractions): (99.55-X) mol% SnO_2_, 0.05 mol% Co_3_O_4_, 0.05 mol% Cr_2_O_3_, 0.35 mol% Nb_2_O_5_, and Xmol% B_2_O_3_ (X = 0.05, 0.15, 0.25, 0.35) for raw material configuration. The specimen preparation process adopted the industrial standardized production process. The specific preparation process is as follows: To a certain proportion of SnO_2_ powder in a ball mill, the appropriate amount of deionized water was added for dispersion ball milling for 4 h. Then, the modified additives Co_3_O_4_, Cr_2_O_3_, Nb_2_O_5_, B_2_O_3_, and so on were added to the ball mill at the same time as an appropriate amount of deionized water, PVA (polyvinyl alcohol), and dispersant (sodium polyacrylate NaPAC), and ball milling continued for 4 h. The dispersed ball-milled mixed slurry was placed in the spray pelletizing tower for spray pelletizing.

**Figure 1:**
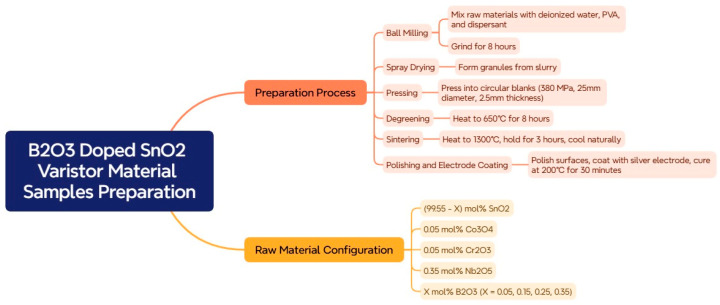
Tin oxide varistor production process flow chart.

The spray-granulated material was placed in a circular mold, and 380 MPa pressure was applied to press it into a circular billet with a diameter of 25 mm and a thickness of 2.5 mm. The pressed embryo was placed in a tunnel furnace, adhesive stripping was performed at a temperature of 650 °C for 8 h, and then it was transferred to a high-temperature furnace (NaberthermLH60/14). The sintering temperature was 1300 °C, the holding time was 3 h, and the heating rate was 5 °C/min. After natural cooling to room temperature, the desired SnO_2_ varistor sample was obtained. The upper and lower surfaces of the sintered cylindrical SnO_2_ varistor samples were polished, and then the surfaces were coated with silver electrodes and cured at 200 °C for 30 min to obtain the SnO_2_ varistor samples that were used for subsequent testing. The process flow diagram is shown in Figure 1.

## 3. Sample Testing

In order to study the electrical characteristics of SnO_2_ varistor samples, the electrical parameters and micromorphology were measured and observed. The microstructures of the sample sections were observed using a scanning electron microscope (Hitachi8010, Hitachi, Chiyoda, Japan), and the average grain size of the SnO_2_ varistor samples was obtained from the measured SEM (Scanning electron microscope) images using the intercept method, which is given by Equation (1) [13,14,15,17,18,19].

(1) $$d=\frac{1.56L}{MN}$$

In Equation (1), L is the length of the reference line measured in the SEM image; M is the magnification of the SEM photo; N is the number of SnO_2_ grains contained in the reference line.

SnO_2_ *I*–*V* characteristics of the varistor samples were measured by a digital source meter (Model 2410, AprilAire, Madison, WI, USA), the value of the voltage gradient was the voltage across the varistor when 1 mA DC current flowed through the varistor, and the leakage current was the value of the current density corresponding to 0.75 V_1 mA_. The nonlinear coefficient α Equation (2) was used for calculations [20].

(2) $$\alpha =\frac{1}{\log E_{2}-\log E_{1}}$$

In Equation (2), $E_{2}$ and $E_{1}$ are the voltage values at a current density of 1 mA/cm^2^ and 0.1 mA/cm^2^ flowing through the varistor, respectively.

The measurement of the *C*–*V* characteristics of piezoresistive materials and the calculation of related data such as barrier height, donor density, and acceptor density involved a broadband dielectric spectrometer (Novocontrol Concept 80, Novocontrol, Montabaur, Germany) [21].

(3) $${\left(\frac{1}{C_{b}}-\frac{1}{2C_{b0}}\right)}^{2}=\frac{2\left(\varphi_{b}+U_{gb}\right)}{q\varepsilon_{r}\varepsilon_{0}N_{d}}$$

In Equation (3), $C_{b0}$ is the capacitance per unit area without bias voltage; $C_{b}$ is the capacitance per unit area without bias voltage; $U_{gb}$ is the bias voltage on the single grain boundary; q is the electronic charge; $\varepsilon_{r}$ is the relative dielectric constant of the SnO_2_ varistor; $N_{d}$ is the donor density; $\varphi_{b}$ is the Schottky barrier height; $\varphi_{b}$ and $N_{d}$ are obtained from the intercept and slope of the *C*–*V* curve.

The relationship between the barrier height $\varphi_{b}$, the donor density $N_{d}$, and the interfacial state density $N_{i}$ is shown in Equation (4), where $\varepsilon_{0}$ is the vacuum dielectric constant.

(4) $$N_{i}={\left(\frac{2N_{d}\varepsilon_{r}\varepsilon_{0}\varphi_{b}}{q}\right)}^{\frac{1}{2}}$$

In order to analyze the thermal behavior of the samples, the heat flux (Q) was measured using a DRL thermal conductivity meter (Xiangtan, China) based on the theory of the heat flow method [22]. The thermal conductivity coefficient K was calculated using Equation (5) [23]:

(5) $$K=\frac{QL}{AT}$$

where the L is the thickness of the sample, the A is the area of the sample, and the T is the temperature difference between the cold and hot sides of the sample. The porosity P of the sample was measured by Archimedes method and calculated as [24]

(6) $$P=\frac{\left(\rho_{th}-\rho_{pr}\right)}{\rho_{th}}\times 100\%$$

In Equation (6), $\rho_{pr}$ is the actual density, and $\rho_{th}$ is the theoretical density [18].

The presence of defects such as point defects, grain boundaries, and dislocations affect the mean free range of phonons. The mean free path of a phonon can be described by the equation

(7) $$\frac{1}{l\left(\omega ,T\right)}=\frac{1}{l_{i}\left(\omega ,T\right)}+\frac{1}{l_{p}\left(\omega \right)}+\frac{1}{l_{b}}$$

$l_{i}\left(\omega ,T\right)$ is the phonon–phonon mean free range; $l_{p}\left(\omega \right)$ is the mean free range reduced by scattering from point defects; $l_{b}$ is the mean free range reduced by grain boundary scattering [23].

## 4. Experimental Results

### 4.1. Microstructure

**Figure 2:**
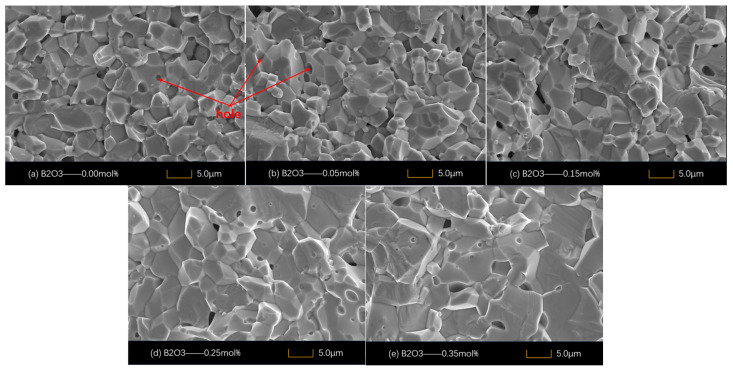
Scanning electron microscope images of different B_2_O_3_ contents in SnO_2_.

Scanning electron microscope images (SEM) of SnO_2_ varistor samples prepared with different doping concentrations are shown in Figure 2a–e. The average grain size d of the samples is shown in Table 1. The cross-sectional microstructures of SnO_2_ varistor samples without the addition of B_2_O_3_ and varistor samples doped with 0.05 mol% B_2_O_3_ are shown in Figure 2a,b, respectively. A large number of air gaps could be seen in the samples, and the grain sizes were uneven.

When the addition amounts of B_2_O_3_ were 0.15 mol% and 0.25 mol%, the grain sizes of the varistor samples increased significantly compared with those of the undoped samples, and the porosity of the samples decreased. This indicated that a small amount of B_2_O_3_ could improve the uniformity and density of the samples. At the same time, due to the melting of low-melting-point B_2_O_3_ into a liquid state during high-temperature sintering, the flowability of SnO_2_ grains was promoted, the liquid-phase sintering time was prolonged, and it was easier to grow. The extension of the liquid-phase sintering time resulted in a more uniform distribution of the modifiers Co_3_O_4_, Cr_2_O_3_, and Nb_2_O_5_ in the sample.

The ionic radius of B^3+^ (0.23 Å) was much smaller than that of Sn^4+^ (0.69 Å), so during the sintering process, B_2_O_3_ might have reacted with SnO_2_ grains in the solid solution, i.e., B^3+^ replaced the position of Sn^4+^ to produce host defects within the SnO_2_ grains, as described in Equation (8), and the resulting oxygen vacancies accelerated the mass transfer of SnO_2_, which facilitated the sintering of SnO_2_, thus making the resulting SnO_2_ samples denser [25].

(8) $$B_{2}O_{3}\overset{SnO_{2}}{\to }2B_{Sn}^{\prime }+V_{O}^{\cdot \cdot }+{3O}_{O}^{X}$$

With the added amount of B_2_O_3_ being 0.35 mol%, the porosity of the sample increased because the excess B_2_O_3_ could not enter the interior of the grains, so it segregated at the grain gaps, resulting in a decrease in density at the grain gaps.

Additionally, abnormal grains can be observed in Figure 2e, which reduced the uniformity of the sample. During the sintering process, the reaction shown in Equation (9) might have occurred, and a small amount of oxygen overflow was generated in the sample, leaving pores and increasing the porosity of this sample.

(9) $$B_{2}O_{3}\overset{1300\;{}^{\circ}C}{\to }2BO+\frac{1}{2}O_{2}\left(g\right)↑$$

**Figure 3:**
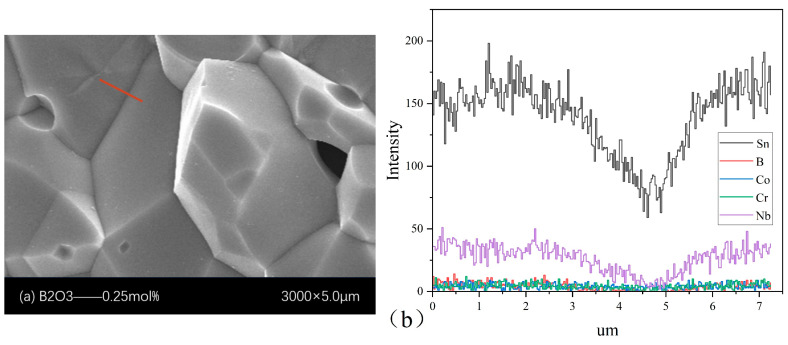

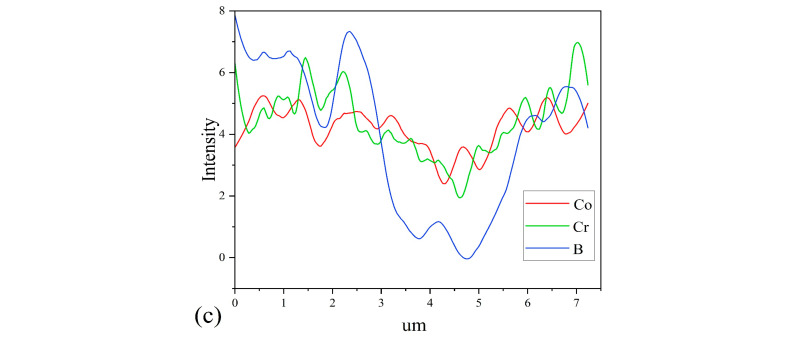
(**a**) SEM microstructure (The red line is the EDS scanning position). (**b**) Schematic of the distribution of the elements in the orange straight line position of the EDS line sweep in the microstructure. (**c**) EDS scan elemental distribution of Co, Cr, and B elements.

Figure 3 shows the variation in elemental content in the EDS line scan. The straight line in Figure 3a represents the EDS line scan position, and Figure 3b shows the curve of the elemental content variation with position. When the line scanning distance is 4.68 μm, the content of all elements significantly decreases, indicating that 4.68 μm is the boundary between two grains. Due to the significantly higher content of Sn and Nb in the sample compared to other elements, the B, Co, and Cr content curves were smoothed for ease of observation, as shown in Figure 3c. It could be observed that Co and Cr were evenly distributed at both the grain and grain boundaries, with the Co and Cr contents being slightly lower at the grain boundaries than at the grains, indicating that Co and Cr mainly entered the lattice positions where they replaced Sn. The processes in Equations (10)–(14) had occurred.

(10) $$Cr_{2}O_{3}\overset{SnO_{2}}{\to }2{Cr}_{Sn}^{\prime }+V_{O}^{\cdot \cdot }+{3O}_{O}^{X}$$

(11) $$2Nb_{2}O_{5}\overset{SnO_{2}}{\to }4Nb_{Sn}^{\cdot }+V_{Sn}^{⁗}+10O_{O}^{X}$$

(12) $$Co_{3}O_{4}\overset{1300\;{}^{\circ}C}{\to }CoO+Co_{2}O_{3}$$

(13) $$Co_{2}O_{3}\overset{SnO_{2}}{\to }2{Co}_{Sn}^{\prime }+V_{O}^{\cdot \cdot }+3O_{O}^{X}$$

(14) $$CoO\overset{SnO_{2}}{\to }{Co}_{Sn}^{\prime \prime }+V_{O}^{\cdot \cdot }+O_{O}^{X}$$

However, B was predominantly present in the grains, suggesting that B entered the grains during the sintering and took the place of Sn, which proved that the process in Equation (8) took place.

**Figure 4:**
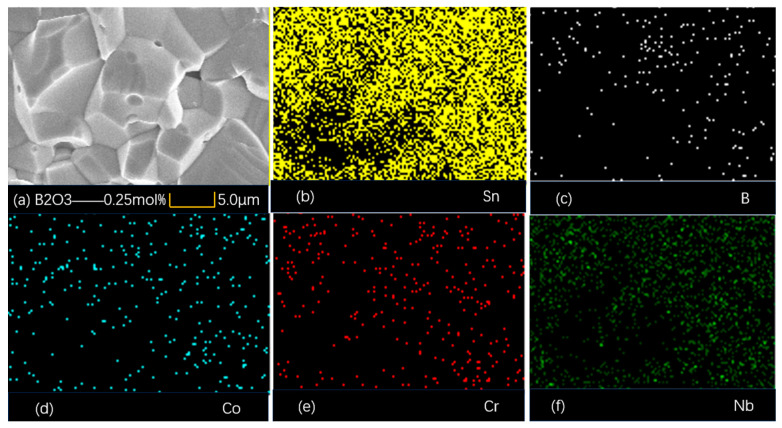
EDS elemental distribution map.

The elemental surface EDS analysis of the specimens with the B_2_O_3_ addition of 0.25 mol% is shown in Figure 4, and the results indicate that B^3+^ ions are doped into the SnO_2_ lattice. The addition of B_2_O_3_ promotes a uniform distribution of grains and a densified microstructure, which results in a grain size of 7.4 μm and a reduction in porosity to 4.2%, as shown in Table 1.

### 4.2. Electrical Characteristics

**Figure 5:**
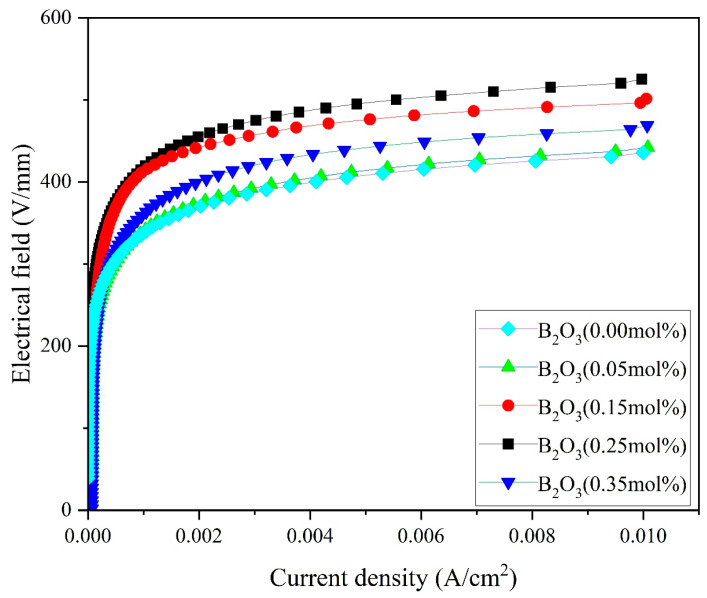
*I*–*V* curves of SnO_2_ samples doped with different amounts of B_2_O_3._

**Figure 6:**
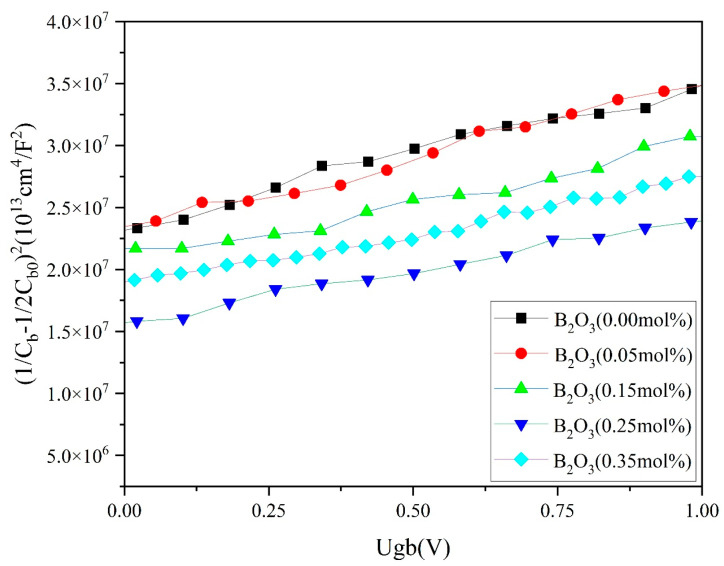
Capacitance–voltage (*C–V*) characteristics of samples doped with different contents of B_2_O_3._

Figure 5 and Figure 6 show the *I–V* and *C–V* curves of the samples, and the voltage gradient *E*_1 mA_, nonlinearity coefficient α, leakage current *J_L_*, donor concentration $N_{d}$, interfacial state density $N_{i}$, and the potential barrier height $\varphi_{b}$ of the varistor were calculated from Equations (2)–(4) and listed in Table 1.

As could be derived from the data in Figure 5, with the increase in the B_2_O_3_ addition in the SnO_2_ varistor from 0.00 mol% to 0.35 mol%, as shown in Table 1, the voltage gradient of the varistor first increased and then decreased with the doping concentration. When the B_2_O_3_ doping concentration was 0.25 mol%, the voltage gradient of the varistor sample reached a maximum of 525 V/mm. At this concentration, the leakage current was minimized to 11.2 μA/cm^2^, and the nonlinear coefficient was maximized to 36.

The doping of B_2_O_3_ in SnO_2_ varistors reacted as shown in Equations (8) and (9), and the doping of Co_3_O_4_, B_2_O_3_, Cr_2_O_3_, and Nb_2_O_5_ introduced a large number of defects such as ${Co}_{Sn}^{\prime \prime },\;{Co}_{Sn}^{\prime }$, ${Cr}_{Sn}^{\prime }$, $V_{Sn}^{⁗}$, $V_{O}^{\cdot \cdot }$, $Nb_{Sn}^{\cdot }$ in Equations (10)–(14) [26,27].

A large number of oxygen vacancies were generated during the reaction process, which easily ionized free electrons and enhanced the N-type semiconductor properties of the SnO_2_ material. Therefore, the introduction of impurities and defects to the SnO_2_ semiconductor doped with Co_3_O_4_, Nb_2_O_3_, and Cr_2_O_3_ increased the nonlinear coefficient of the sample.

Figure 6 shows the capacitance–voltage (*C–V*) characteristics of the samples with different B_2_O_3_ contents. The $N_{d}$, $\varphi_{b}$, and $N_{i}$ of samples could be obtained from the characteristic curves by Equations (3) and (4), and the results are summarized in Table 1. It can be seen from Table 1 that the $N_{i}$, $N_{d}$, and $\varphi_{b}$ all showed a trend of increasing and then decreasing with the gradual increase in the B_2_O_3_ doping concentration. Equation (8) shows that a portion of B^3+^ reacted with Sn^4+^ in a defective way to produce $B_{Sn}^{\prime }$, which contributed to the formation of Schottky barriers. B_2_O_3_ as an acceptor dopant increased the hole concentration in the sample, resulting in an increase in *N*_d_. The change in $N_{i}$ was due to the doping of low-melting-point B_2_O_3_, which lowered the starting temperature of liquid-phase sintering, prolonged the sintering time of the liquid phase, promoted oxygen transfer, and increased the oxygen content in the sample.

According to Equation (4), it could be seen that when the increase in the interface state concentration was greater than the donor density, the width of the depletion layer could be widened, leading to an increase in the grain boundary potential barrier. When the doping concentration of B_2_O_3_ was 0.25%, the maximum value of $\varphi_{b}$ was 1.24 eV. This was because a small amount of B_2_O_3_ doping promoted ion exchange between the grains and other additives at the grain boundaries in the varistor, which helped to increase the barrier height and reduce leakage current. When the doping concentration of B_2_O_3_ was 0.35 mol%, both the voltage gradient and nonlinear coefficient decreased because the increase in grain size led to a decrease in the number of unit grain boundaries, resulting in a decrease in grain boundary potential barriers and voltage gradients.

### 4.3. Thermal Conductivity

When the doping concentration of B_2_O_3_ increased from 0.00 mol% to 0.15 mol%, the thermal conductivity also increased from 4.88 W/m·K to 5.02 W/m·K. When the doping concentration of B_2_O_3_ was 0.25 mol%, the maximum thermal conductivity was 6.13 W/m·K, and as the doping concentration further increased, the thermal conductivity decreased to 5.93 W/m·K.

According to Equation (7), the presence of defects such as point defects, grain boundaries, and dislocations increased the number of phonons, increased the probability of phonon collisions, further enhanced phonon scattering, changed the initial heat transfer direction of phonons, and released their energy. At the same time, collisions caused energy loss and weakened or terminated phonon transmission, resulting in a decrease in the mean free path of phonons in the varistor samples and a decrease in thermal conductivity efficiency. Phonon scattering was inversely proportional to thermal conductivity efficiency.

When the grain size increased, the number of grain boundaries through which heat transfer paths passed decreased, phonon scattering at grain boundaries decreased, and thermal conductivity efficiency increased accordingly. Based on the microstructure data of the sample in Table 1 and the variation in thermal conductivity in Figure 6, it could be concluded that as the B_2_O_3_ doping concentration in the sample increased from 0.00 mol% to 0.25 mol%, the grain size of the sample increased from 6.8 μm to 7.4 μm. Therefore, in the process of heat transfer, the number of grain boundaries traversed by the heat transfer path decreased as the grain size increased. In Equation (7), $l_{b}$ decreased with increasing doping concentration, resulting in a decrease in phonon scattering and an increase in thermal conductivity.

Phonon scattering occurred through the pores, and excessive porosity increased the energy loss in the phonon-scattering process, thus reducing the overall thermal conductivity of the sample [28,29]. When the doping concentration of B_2_O_3_ was increased to 0.35 mol%, the appearance of anomalous grains could be observed from Figure 2d, and the homogeneity of the sample was reduced. At the same time, too much B_2_O_3_ occurred as in Equation (9), and the resulting oxygen overflowed to form air holes during the sintering process, which reduced the densification of the sample and ultimately led to an increase in the porosity of the sample. The existence of pores made the number of phonons increase during the heat conduction process, and the probability of phonon collision increased, which increased the phonon scattering, i.e., $l_{p}\left(\omega \right)$ in Equation (7) increased, which ultimately led to a decrease in the thermal conductivity of the sample, thus adversely affecting the heat dissipation of the varistor.

**Figure 7:**
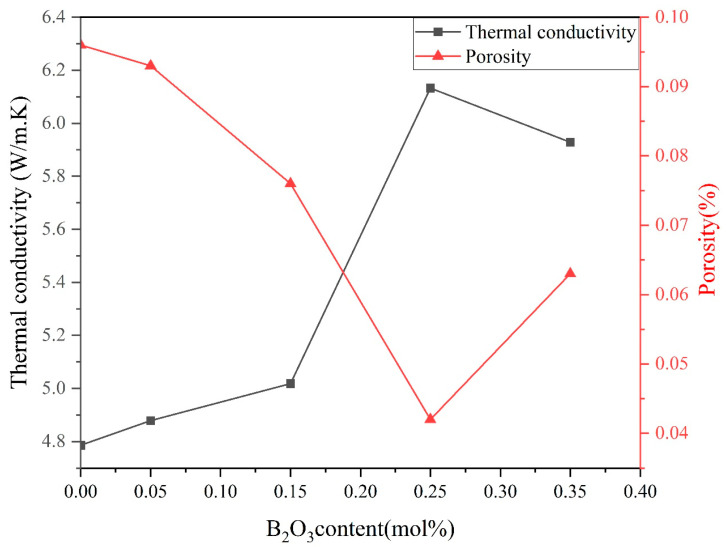
Curves of SnO_2_ porosity and thermal conductivity efficiency with B_2_O_3_ doping at fixed temperature.

From Figure 7, it can be obtained that the porosity of the varistor samples was inversely proportional to the thermal conductivity when B_2_O_3_ doping was increased from 0.00 mol% to 0.35 mol%. Additionally, it can be concluded that the main factor affecting the thermal conductivity under this experimental formulation was the porosity.

The varistor had the lowest porosity and the highest thermal conductivity when B_2_O_3_ doping was 0.25 mol%, which meant that the efficiency of heat transfer of the samples in this formulation was the greatest and had good heat dissipation performance.

**Figure 8:**
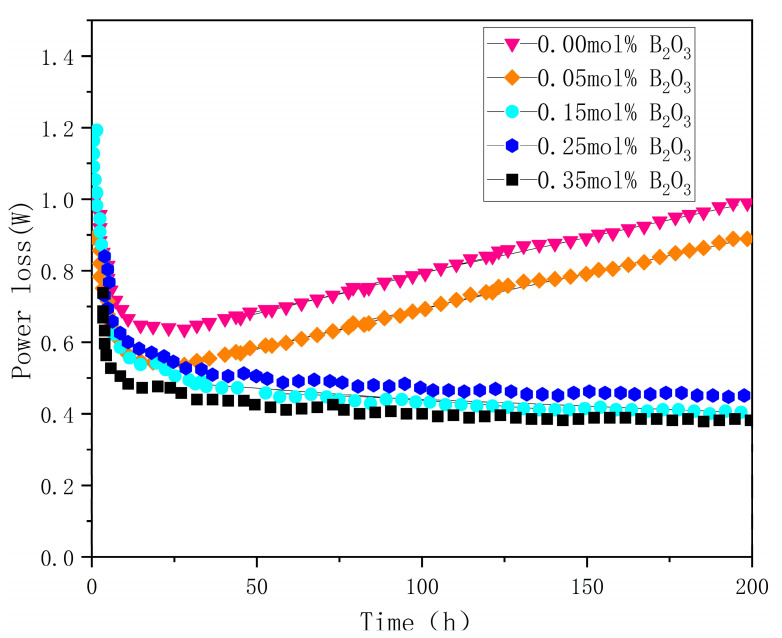
Changes in power loss of samples aged for 200 h.

As depicted in Figure 8, to assess the impact of B_2_O_3_ doping on SnO_2_ varistors, 200 h DC accelerated aging tests were performed on samples with doping levels of 0, 0.05, 0.15, 0.25, and 0.35 mol%. The results indicated that samples doped with 0 and 0.05 mol% exhibited a gradual increase in power loss over time. This was attributed to the high leakage current density, which induced heating in the varistor ceramics under DC bias. Given their negative temperature coefficient, leakage currents escalated, leading to thermal runaway. In contrast, samples doped with 0.15, 0.25, and 0.35 mol% showed an initial peak in power loss, followed by a gradual decrease to a stable value as time progressed. This stabilization resulted from an increased barrier height, primarily caused by the realignment of ions at grain boundaries under the applied voltage, which broadened the depletion layer. These findings demonstrated that appropriate B_2_O_3_ doping significantly improved the aging stability of SnO_2_ varistors.

## 5. Conclusions

This study systematically investigated the synergistic optimization mechanism of B_2_O_3_ doping on the electrical properties and thermal conductivity of SnO_2_-Co_3_O_4_-Cr_2_O_3_-Nb_2_O_5_-B_2_O_3_ varistors. The experimental results demonstrated that the sample with 0.25 mol% B_2_O_3_ exhibited optimal comprehensive performance: a voltage gradient of 525 V/mm, a nonlinear coefficient of 36, a leakage current density as low as 11.2 μA/cm^2^, and a thermal conductivity of 6.13 W·m^−1^·K^−1^. Microstructural analysis revealed that moderate B_2_O_3_ doping facilitated grain densification and liquid-phase sintering homogeneity through solid solution substitution of Sn^4^⁺ (generating oxygen vacancy defects), which significantly reduced porosity (4.2%) and increased grain size (7.4 μm). The simultaneous enhancement of grain boundary barrier height (φb = 1.24 eV) and interfacial state density (Ni = 6.8 × 10^15^ m^−2^) effectively suppressed leakage currents and strengthened nonlinear responses. Furthermore, grain coarsening reduced phonon-scattering paths, while low porosity decreased thermal resistance, synergistically improving thermal conductivity by 25.6% compared to undoped samples. This study provides a theoretical foundation for developing SnO_2_-based varistors with high electrical performance and thermal stability, which can be extended to the insulation design optimization of surge arresters in ultra-high-voltage power grids. Future work should explore long-term aging behavior under multi-field coupling (e.g., temperature–electric field–mechanical stress) and validate process reproducibility in industrial-scale production.

## Figures and Tables

**Table 1:** Microstructure and electrical properties of SnO_2_ with different B_2_O_3_ doping levels.

B_2_O_3_ (mol%)	Porosity %	d (μm)	E_1 mA_ (V/mm)	J_L_ (μA/cm^2^)	α	N_d_ (10^22^ m^−2^)	N_i_ (10^15^ m^−2^)	Φb (eV)
0.00	9.4%	6.7	421	25	26	1.8	3.0	0.82
0.05	9.3%	6.8	448	23	29	1.9	3.2	0.85
0.15	7.6%	7.2	501	18.3	31	2.6	4.4	0.98
0.25	4.2%	7.4	525	11.2	36	4.7	6.8	1.24
0.35	6.3%	8.6	428	14.6	33	3.6	5.4	1.15

## Data Availability

The original contributions presented in this study are included in the article. Further inquiries can be directed to the corresponding author.
